# Discovery and Genome Characterization of a Closterovirus from Wheat Plants with Yellowing Leaf Symptoms in Japan

**DOI:** 10.3390/pathogens12030358

**Published:** 2023-02-21

**Authors:** Hideki Kondo, Hitomi Sugahara, Miki Fujita, Kiwamu Hyodo, Ida Bagus Andika, Hiroshi Hisano, Nobuhiro Suzuki

**Affiliations:** 1 Institute of Plant Science and Resources (IPSR), Okayama University, Kurashiki 710-0046, Japan; 2 College of Plant Health and Medicine, Qingdao Agricultural University, Qingdao 266109, China

**Keywords:** wheat, closterovirus, yellow leaf disease, RNA-seq, aphid transmission, RNA silencing, Japan

## Abstract

Many aphid-borne viruses are important pathogens that affect wheat crops worldwide. An aphid-transmitted closterovirus named wheat yellow leaf virus (WYLV) was found to have infected wheat plants in Japan in the 1970s; however, since then, its viral genome sequence and occurrence in the field have not been investigated. We observed yellowing leaves in the 2018/2019 winter wheat-growing season in an experimental field in Japan where WYLV was detected five decades ago. A virome analysis of those yellow leaf samples lead to the discovery of a closterovirus together with a luteovirus (barley yellow dwarf virus PAV variant IIIa). The complete genomic sequence of this closterovirus, named wheat closterovirus 1 isolate WL19a (WhCV1-WL19a), consisted of 15,452 nucleotides harboring nine open reading frames. Additionally, we identified another WhCV1 isolate, WL20, in a wheat sample from the winter wheat-growing season of 2019/2020. A transmission test indicated that WhCV1-WL20 was able to form typical filamentous particles and transmissible by oat bird-cherry aphid (*Rhopalosiphum pad*). Sequence and phylogenetic analyses showed that WhCV1 was distantly related to members of the genus *Closterovirus* (family *Closteroviridae*), suggesting that the virus represents a novel species in the genus. Furthermore, the characterization of WhCV1-WL19a-derived small RNAs using high-throughput sequencing revealed highly abundant 22-nt-class small RNAs potentially derived from the 3′-terminal end of the WhCV1 negative-strand genomic RNA, indicating that this terminal end of the WhCV1 genome is likely particularly targeted for the synthesis of viral small RNAs in wheat plants. Our results provide further knowledge on closterovirus diversity and pathogenicity and suggest that the impact of WhCV1 on wheat production warrants further investigations.

## 1. Introduction 

Wheat (*Triticum aestivum* L.) is the second most commonly grown cereal crop after maize (*Zea mays* L.) and the source of one of the most important staple foods in the world. Among wheat- and/or barley (*Hordeum vulgare* L.)-infecting viruses, of which at least 57 exist, several vector-borne viruses have been described as important pathogens significantly impacting wheat production [1,2]. The causative agents of yellow dwarf and wheat streak mosaic diseases are likely common globally and threaten wheat production worldwide [3]. Regarding yellow dwarf diseases, members of two different viral genera, *Luteovirus* (family *Tombusviridae*) and *Polerovirus* (family *Solemoviridae*), are known to possess monopartite positive-sense RNA (+RNA) genomes [4]. Both types of RNA viruses are phloem-limited and transmitted in a circulative-persistent manner by aphids (order Hemiptera), such as the bird cherry-oat aphid (*Rhopalosiphum padi*) and corn leaf aphid (*Rhopalosiphum maidis*) [5]. Barley yellow dwarf virus PAV (BYDV-PAV, a luteovirus) is one of the most prevalent and abundant cereal virus species worldwide [5,6].

In Japan, a few aphid-borne viruses, such as BYDV-PAV and cereal yellow dwarf virus PRS (a polerovirus), have been reported to infect wheat and barley plants [7,8,9]. Additionally, an aphid-transmissible (via *R. maidis*) wheat yellow leaf virus (WYLV) with filamentous virions (~1800 nm ×10 nm) associated with leaf yellowing symptoms in wheat and barley plants was reported in Japan in the 1970s [9,10,11]. WYLV is currently classified as a member of the genus *Closterovirus*, but its molecular properties have not yet been characterized [12].

The members of the plant virus family *Closteroviridae* have filamentous virions 650–2200 nanometer (nm) in length and are currently classified into seven genera: *Ampelovirus* (*n* = 13), *Bluvavirus* (*n* = one), *Closterovirus* (*n* = 17), *Crinivirus* (*n* = 14), *Menthavirus* (*n* = one), *Olivavirus* (*n* = three), and *Velarivirus* (*n* = eight) [12]. They have a large +RNA genome of a mono-, bi-, or tripartite nature with lengths of 13,000 to nearly 19,000 nucleotides (nt) [12]. The genome organization of closterovirids is mainly conserved, with two genomic coding blocks [12,13]. The first gene module encodes replication-associated proteins encoded by open reading frames 1a and 1b (ORF1a and 1b). The ORF1b protein is an RNA-directed RNA polymerase (RdRP) that is likely expressed through +1 ribosomal frameshift to fuse with an ORF1a protein [13]. The second module, with conserved quintuple genes, encodes proteins associated with virion assembly and/or cell-to-cell movement [13]. These viruses are transmitted by aphids (closteroviruses), whiteflies (criniviruses), and soft scale insects or pseudococcid mealybugs (ampeloviruses and velariviruses) in a semi-persistent manner, whereas the vectors of bluvaviruses and olivaviruses remain unidentified. Among closterovirids, only a few members of the genus *Closterovirus* are mechanically transmissible, although with low efficiency [12]. Most of the members of the family *Closteroviridae* are pathogens of or associated with dicotyledonous crops, including vegetables and fruit trees. In recent studies, the de novo RNA-sequencing (RNA-seq) approach has enabled researchers to uncover many thus-far-unknown viral genome sequences in the family, including the members or candidates of the genus *Closterovirus* [14,15,16,17,18,19,20,21,22,23,24].

In this study, an RNA-seq approach was used to investigate the viral agent associated with yellow leaf symptoms in wheat plants from an experimental field in Kurashiki, Japan. We discovered two isolates (WL19a and WL20) of a novel closterovirus, tentatively named wheat closterovirus 1 (WhCV1), along with a coinfecting isolate of a BYDV-PAV variant. We also found that WhCV1 could be transmitted via an aphid population (*R. padi*) and targeted by host antiviral RNA silencing. The relationship between WhCV1 and a historical closterovirus (WYLV) previously found in the same field is also discussed here.

## 2. Results

### 2.1. Identification of Virus-Related Sequences from Yellow Leaf Samples of Wheat Plants via RNA-Seq

**Figure 1:**
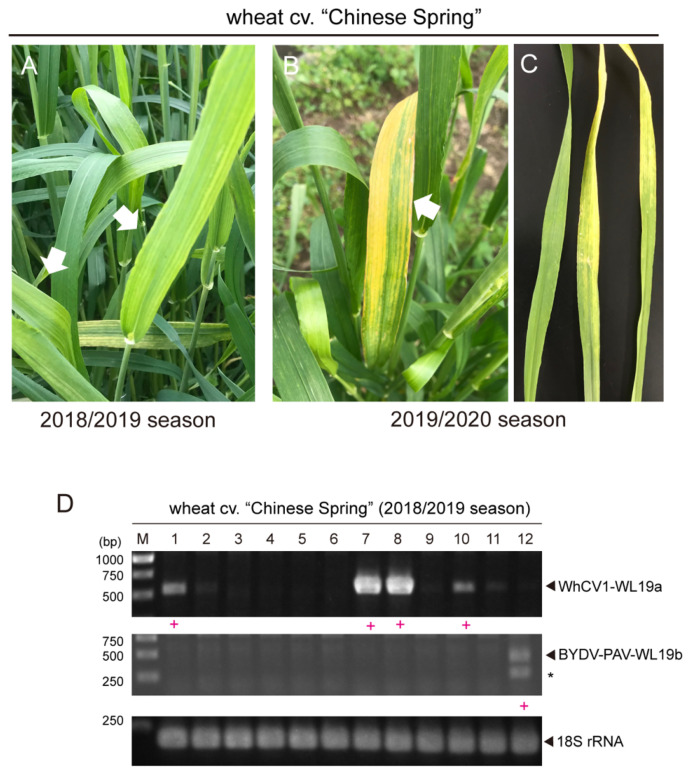
Detection of WhCV1 in wheat grown in the field. (**A**,**B**) Representative yellow leaf symptoms in wheat (cv. “Chinese Spring”) in the experimental field during the winter wheat-growing season of 2018/2019 ((**A**), early April) and 2019/2020 ((**B**), mid-April). White arrows indicate symptomatic wheat leaves. (**C**) The detached wheat symptomatic leaf samples from the 2019/2020 season subjected to the vector transmission test (left, an asymptomatic leaf sample was included as a control). (**D**) RT-PCR detection of WhCV1 from wheat leaf samples (mid-May, growing season 2018/2019). M, 1-kb DNA ladder (Thermo Fisher Scientific, Waltham, MA, USA) used as a size marker. Samples in which a closterovirus (WhCV1-WL19a) or a luteovirus (BYDV-PAV) was detected are indicated by a red plus sign (+) below the gel lane. Asterisks indicated unknown amplicons.

**Figure 2:**
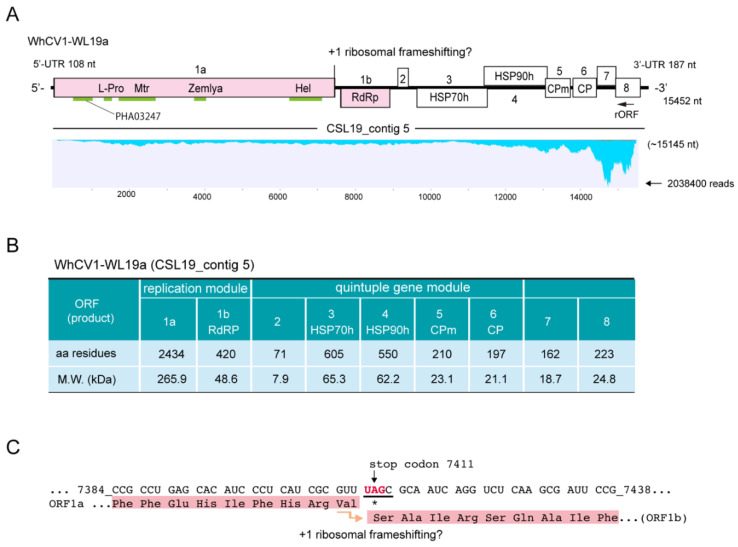
Characteristics of the WhCV1 genome and proteins. (**A**) Genome structure of the WhCV1-WL19a isolate (CSL19_contig 5) obtained from RNA-seq of the wheat leaf sample (winter wheat-growing season of 2018/2019). The conserved domains, methyltransferase (Mtr), helicase (Hel), RNA-dependent RNA polymerase (RdRP), leader protease (L-Pro), Zemla (see main text), and PHA03247 domains are indicated with light green lines. The ORF8 protein has an RSS_P20 superfamily domain (pfam11757, E-value = 5e^−07^; not indicated). HSP70h: heat shock protein 70 homolog; HSP90h: ~60 kDa protein; CPm: minor coat protein; CP: coat protein. Note that a small reverse ORF (rORF, 525 nt in length) overlapping the ORF8 region was also predicted (no BLAST-P hits). The putative WhCV1-derived sequence reads were mapped to the genome using the Read Mapping algorithm of the CLC Genomics Workbench (parameters: length fraction = 0.75; similarity fraction = 0.95). The y-axis indicates the mapped-read coverage with the maximum read number. (**B**) The properties of the predicted coding proteins of the WhCV1-WL19a isolate. (**C**) The putative frameshift site of the replicase (ORF1a-1b fusion protein). Numbers refer to the start and end position of the nucleotide sequences of the WhCV1-WL19a genome and nucleotide sequences related to the frameshift are underlined.

After de novo assembly, a total of 81,494 sequence contigs were obtained through RNA-seq of the RNA samples extracted from symptomatic wheat leaves that were collected during the 2018/2019 winter wheat-growing season (collected in April 2019; Figure 1A). Among them, two virus-like sequence contigs, namely CS L19_contig 5 and CS L19_contig 1289 (15,145 and 5699 nt in length, respectively), were identified to have sequence similarities with closteroviruses and BYDV-PAV isolates, respectively, but no other virus-related contig was identified (Table 1). The 1,995,569 and 37,336 raw reads were mapped onto contig CSL19_contig 5 and CSL19_contig 1289, respectively, using the read mapping algorithm tool of CLC Genomics Workbench (Figure 2A). The presence of two viral candidates (CSL19_contig 5 and CSL19_contig 1289) was verified by reverse transcription-polymerase chain reaction (RT-PCR) analysis using specific primer sets for each of the contigs (Supplementary Table S1). These two viruses were also detected by RT-PCR, and their representative amplicons were sequenced in one or more tested “Chinese Spring” samples randomly collected from the field in mid-May 2019 (Figure 1D), further confirming the presence of these two viruses in the field. However, the association between the presence of these viruses and the yellowing symptoms was unclear because the leaves were collected at the late stage of plant growth (mid-May), when many wheat plants showed leaf senescence (see also below).

### 2.2. Complete Genome Sequence of Closterovirus from the Wheat Yellow Leaf Sample

Based on the CS19L_contig 5 sequence, regions throughout the genome of a closterovirus were amplified by RT-PCR using total RNA samples, and their nucleotide sequences were verified via direct Sanger sequencing of amplicons or cDNA clones. Subsequently, 3′ RNA ligase-mediated rapid amplification of complementary cDNA ends (RLM-RACE) analysis was performed for the 5′- and 3′-terminal regions (primers are listed in Supplementary Table S1). The complete genome sequence of the closterovirus (WL19a isolate) was 15,452 nt in length (accession no. LC735716) and included 108-nt 5′- untranslated region (UTR) and 187-nt 3′-UTR sequences (Figure 2A, upper row). The 3′ terminal of the genomic RNA was lacked a poly(A) tail, similar to other closteroviruses. The sequence showed no significant hits in basic local alignment search tool-nucleotide (BLAST-N) searches against the National Center for Biotechnology Information (NCBI) database; thus, we tentatively named this closteroviral candidate as “wheat closterovirus 1” (WhCV1). The predicted genome organization of WhCV1 consisted of nine ORFs: ORF1a and ORF1b, and ORF2–ORF8 (Figure 2A). The prediction of the RNA secondary structures using the Mfold program showed that the sequences of the 5′- and 3′-UTRs of the positive-strand genome contained some potential stem-and-loop structures (Supplementary Figure S1). In the case of citrus tristeza virus (CTV, a well-characterized closterovirus), stem-and-loop structures in the both 5′-and 3′-UTRs are known to be involved in viral replication or assembly (only for the 5′-UTR) [25,26,27].

### 2.3. Sequence Characteristics of the Predicted WhCV1-Encoded Proteins

WhCV1-WL19a ORF1a and 1b encoded putative replication-associated proteins. ORF1a encoded a protein that had a 34.4% identity to the 278 kDa protein of Rehmannia virus 1, whereas the ORF1b protein had 61.3% identity to the RdRP of soybean leaf crinkle mottle virus (Table 2). The ORF1a protein (2434 amino acids [aa], 265.9 kDa) contained a papain-like cysteine protease (L-Pro) domain and a methyltransferase (Mtr) domain (viral methyltransferase, pfam01660) in the N-terminal region and a helicase (Hel) domain (viral superfamily RNA helicase, pfam01443) in the C-terminal region (Figure 2A,B). Another conserved domain, named the “Zemlya region”, was also predicted to be present between the Mtr and Hel domains (Figure 2A). WhCV1-WL19a ORF1b encoded the RdRP domain (420 aa, viral RNA-directed RNA polymerase, pfam00978) presumably expressed via a +1 ribosomal frameshift from ORF1a, which is a common feature of closterovirids [28,29] (Figure 2A,B). In WhCV1 and other closterovirids, the sequence of the putative +1 slippery site of the first ORF (ORF1a) has the conserved motif “GUU_stop_C”, with some exceptions [14,30] (Figure 2C). The sequence of the putative +1 slippery site (…_7408_GUUUAGC; ORF1a stop codon is underlined) is presented in Figure 2C. The frameshifting translation from the first gene unit (ORF1a/1b) in the WhCV1-WL19a genome would likely be able to produce a large fusion protein of 3197 kDa (2898 aa), forming the common module “Met-Hel-RdRP” of the alphavirus-like superfamily [31].

The second gene unit, a quintuple gene block of WhCV1-WL19a (ORFs 2–6), was predicted to code for a hydrophobic protein (71 aa, 7.9 kDa), a homolog of plant heat shock protein 70 (HSP70h; 605aa, 65.3 kDa), a HSP90 homologue (HSP90h or ~60 kDa protein; 550 aa, 62.2 kDa), as well as a minor coat protein (CPm; 210aa, 23.1kDa) and coat protein (CP; 197 aa, 21.1 kDa) (Figure 2A,B). BLAST searches indicated that the WhCV1-WL19a ORF2 protein showed no significant similarity to other known viral proteins, although it was predicted to contain a transmembrane domain in the C-terminal region (47–70 aa region) like other closteroviral counterparts. The ORF3, ORF4, ORF5, and ORF6 proteins of WhCV1-WL19a had the highest amino acid identities to the HSP70h of Cnidium closterovirus 1 (51.4%), HSP90h of mint virus 1 (43.9%), CPm of beet yellow stunt virus (38.0%) and CP of grapevine leafroll-associated virus 2 (33.5%), respectively (Table 2). The two downstream ORFs (ORFs 7 and 8) encode 18.7-kDa (162 aa) and 24.8-kDa (223 aa) proteins, respectively (Figure 2A,B). A BLAST-P search yielded no significant hits for the ORF7 protein, whereas the ORF8 protein showed a weak sequence similarity to the CTV p20 protein (a viral suppressor of RNA silencing [VSRs]; 25.9%, E-value = 1e^−04^). Taken together, based on the species demarcation criteria for the genus *Closterovirus* (>25% amino acid sequence divergence) [12], these data suggest that WhCV1-WL19a likely represents a novel species in this genus.

### 2.4. Identification of a WhCV1 Variant and Evaluation of Its Aphid Transmission

**Figure 3:**
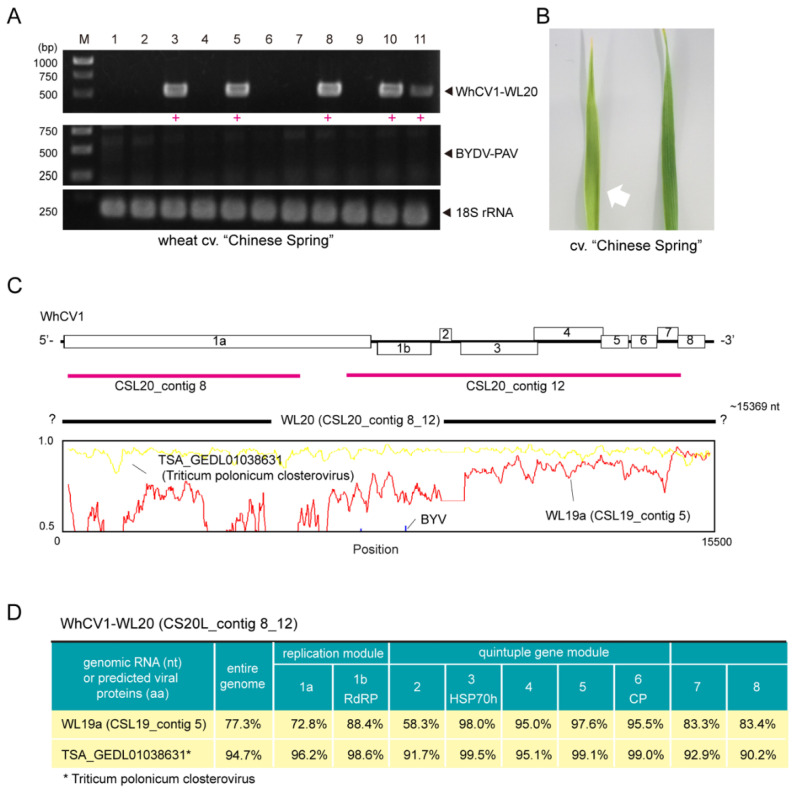
Aphid transmissibility and genome sequence properties of the WhCV1-WL20 isolate obtained from a symptomatic leaf sample from the winter wheat-growing season of 2019/2020. (**A**) RT-PCR detection of WhCV1-WL20 and BYDV-PAV in wheat seedlings 6 weeks after inoculation feeding (right panel). M, 1-kb DNA ladder used as a size marker. Red plus signs (+) indicated samples positive for WhCV1-WL20 or BYDV-PAV. (**B**) A leaf of wheat cv. “Chinese Spring” observed with an yellowing symptom 5 weeks after aphid feeding inoculation (left-hand side with a white arrow) together with a leaf of the mock inoculation plant (right-hand side). (**C**) The nucleotide sequence similarity plot of the entire genome of the WhCV1-WL20 isolate (CSL20_contig 8_12) with the WL19a isolate (CSL19_contig 5, red line), potential WhCV1 variant (TSA_GEDL01038631, named Triticum polonicum closterovirus, yellow line), and a known closterovirus (beet yellows virus [BYV], blue line; NC_001598). The plot was generated using Simplot v. 3.5.1. The y-axis indicates the percent identity using a 200-nt-wide sliding window with a 20-nt step size. The x-axis shows the base position along the genome. Two originally assembled contigs derived from the WL19a isolate (CSL20_contigs 8 and 12) are indicated by thick red bars. (**D**) Nucleotide and amino-acid sequence identities of WhCV1 variants.

In April of the 2019/2020 winter wheat-growing season, we observed the yellow leaf symptoms on cv. “Chinese Spring” plants in the Institute of Plant Science and Resources (IPSR) field (Figure 1B). RT-PCR detection of WhCV1 was then performed using original primer sets for WhCV1-WL19a and BYDV-PAV (Supplementary Table S1); however, these viruses were not detected. After using some other primer sets designed based on an unannotated transcriptome shotgun assembly (TSA) sequence related to WhCV1 (accession no. GEDL01038361, see below), infections of a putative variant of WhCV1 were detected (data not shown, see below). Thereafter, a specific primer set for this WhCV1 variant (named WL20) was designed (Supplementary Table S1). To evaluate aphid transmissibility, a virus-free adult aphid *R. padi* population confirmed by RNA-seq analysis (H. Kondo, unpublished results) was placed on WhCV1-WL20-infected symptomatic leaves for 48 h to allow for virus acquisition (Figure 1C). After a 5-day inoculation access period on virus-free wheat seedlings grown in a plant growth chamber, systemic leaves of inoculated plants were tested for WhCV1-WL20 infection by RT-PCR 6 weeks post inoculation (Supplementary Table S1). Of 11 tested wheat plants, five individual plants of “Chinese Spring” were positive for WhCV1-WL20 (GEDL01038361-related), whereas neither BYDV-PAV nor WhCV1-WL19a isolate was detected (Figure 3A, data not shown). Similar results were obtained with wheat cv. “Kitahonami” via a viruliferous aphid population (data not shown). Virus-like filamentous particles were detected in these leaf samples (Supplementary Figure S2). WhCV1-WL20-infected plants showed mild yellowing leaf symptoms, although the symptoms appeared not to be persistent throughout the plants’ growth, as newly developed leaves or shoots often showed no symptom (Figure 3B and data not shown). It is unclear whether an experimental condition in the plant growth chamber affected WhCV1 symptom developments, therefore, this should be investigated further in the future.

One of the wheat infectants was selected to maintained the virus through passage via aphid transmissions in the same growth chamber as an established laboratory WhCV1 isolate (WL20 isolate). Since this isolate was likely to have a slightly different genome sequence from that of WhCVI-WL19a, we conducted an rRNA-depleted RNA-seq analysis using this plant to determine the genome sequence of the WhCV1-WL20 isolate. After de novo assembly and subsequent local BLAST analysis, two closterovirus-related contigs (CSL20_contig 8: 5780 nt and CSL20_contig 11: 7965 nt, respectively) were obtained, which covered around 30% and 51% of the genomic regions, respectively (Figure 3C, red line); no other viral candidate was identified in this sample. A sequence gap from the two assembled contigs was determined by a gap-filling RT-PCR, and the sequence of the entire genome was validated with overlapping RT-PCR followed by Sanger sequencing as well as 3′ RLM-RACE (the amplifications of the 5′-terminal were not successful). The nearly-complete genomic sequence of the WhCV1-WL20 isolate was 15,369 nt in length (named CSL20 contig 8_11; total mapped read number, 8,616,360; accession no., LC735717; Figure 3C, black line). The constructed sequence is likely missing 13-nt of the 5′-terminal sequences, and the 5′ half of the genome was more divergent than the remaining 3′ region (Figure 3C, lower row). Accordingly, pairwise protein similarity analyses of the two WhCV1 isolates showed that the predicted proteins of the first gene module located in the 5′ region (ORF1a and 1b proteins) were less conserved (72.8% and 88.4% identity, respectively) than those of the quintuple gene module in the 3′ part of the genome (HSP70h, HSP90h, CPm, and CP), which had around 95.0–98.0% identities, except for a short ORF2 protein that had 58.3% identity (Figure 3D). The remaining two ORF (ORF7 and 8) in the 3′ region showed intermediate values.

While mining the public transcriptome database for the discovery of novel viral sequences [32], we discovered the presence of an unannotated TSA sequence related to WhCV1 (accession no. GEDL01038361, 15,366 nt in length) in the transcriptomic data of Polish wheat (*Triticum polonicum* L.) from a Chinese research group [33]. During the preparation and submission of this paper, another research group mined the same TSA sequence for the same virus as we discovered and tentatively named it Triticum polonicum closterovirus (TriPCV; TPA no. BK059767) [34]. The Polish wheat TSA sequence covered almost the entire genomic region of WhCV1 and showed high sequence similarities with the WL20 isolate (CSL20_contig 8_12) regarding nucleotide (94.7% identity) and amino acid (90.2–99.5% identity) sequences (Figure 3C,D).

### 2.5. Similarity and Phylogenetic Relationship of WhCV1 and Other Closteroviruses

**Figure 4:**
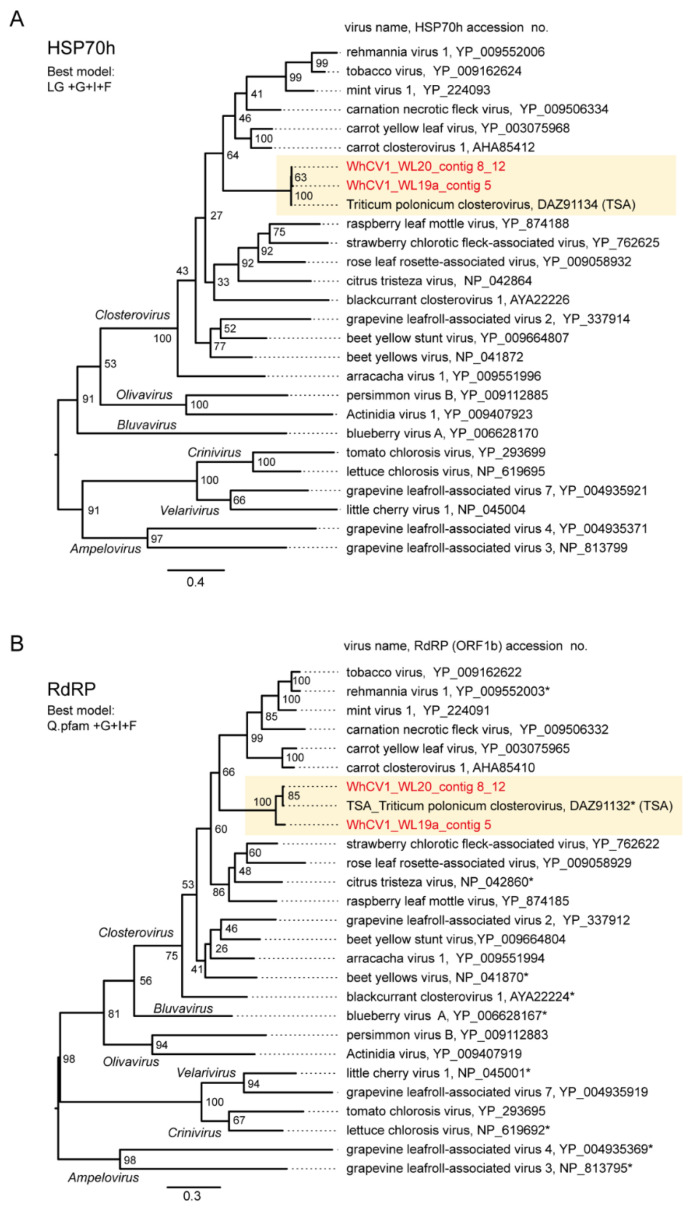

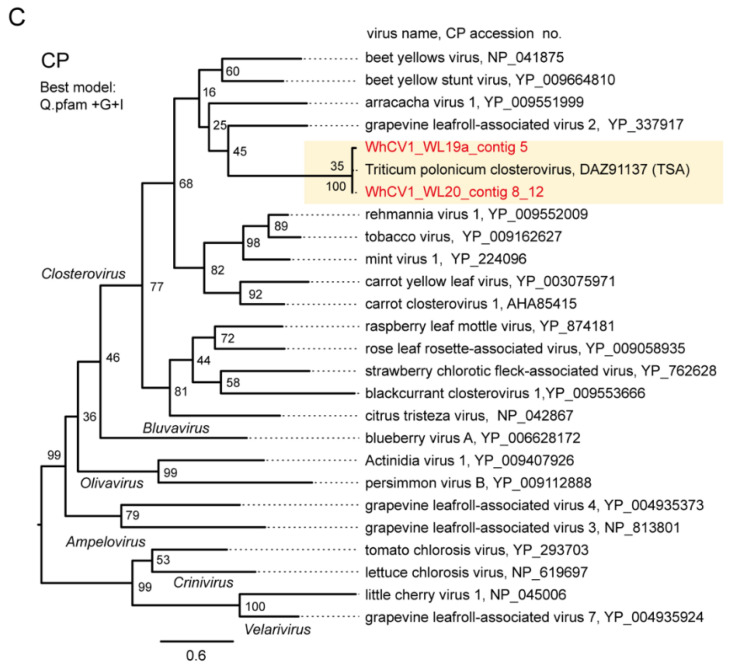
Phylogenetic relationships of the novel wheat closterovirus. (**A**–**C**) Phylogenetic trees reconstructed using the HSP70h (**A**), RdRP (ORF1b protein) (**B**), and CP (ORF6 protein) sequences (**C**) of the representative members of the genus *Closterovirus.* Maximum likelihood (ML) trees were constructed using a MAFFT alignment of the HSP70h, RdRP or CP amino acid sequences. The virus names referring to plant viruses are followed by the GenBank accession numbers or reference sequence (Refseq) numbers of their sequences. WhCV1 isolates were shown in red (obtained from the current study) and highlighted with a yellow background. Representative members of the viral species were used for the analyses. A part of the polyprotein (1a-1b fusion) entries is marked with asterisks following the accession numbers. The members of related virus groups (genera *Ampelovirus, Crinivirus, Velarivirus, Bluvavirus*, and *Olivavirus*) were used as the outgroups. The statistical significance of branches was evaluated with bootstrap analysis (1000 iterations). Bars represent amino acid divergence.

Pairwise sequence identity analyses using three key proteins HSP70h (ORF3 protein), replicase (RdRP), and CP, encoded by closteroviruses were conducted. The WhCV1 HSP70h, replicase and CP candidates showed moderate to low levels of amino acid identity with the corresponding proteins of other closteroviruses (HSP70h: 39–52%; RdRP: 52–60%, and CP: 19–29%; Supplementary Figure S3). To examine phylogenetic relationships between WhCV1 and other closteroviruses, we generated maximum likelihood (ML) phylogenetic trees based on the amino acid alignments of their HSP70h, RdRP, and CP, like in the above pairwise identity analyses. In the ML trees of HSP70h, RdRP, and CP, WhCV1 formed a strong or moderately supported large monophyletic clade with other closteroviruses (Figure 4). Notably, although WhCV1 isolates formed a clade within the genus *Closterovirus*, they were distantly related to members of other established species.

### 2.6. Small RNA Profiles of WhCV1

**Figure 5:**
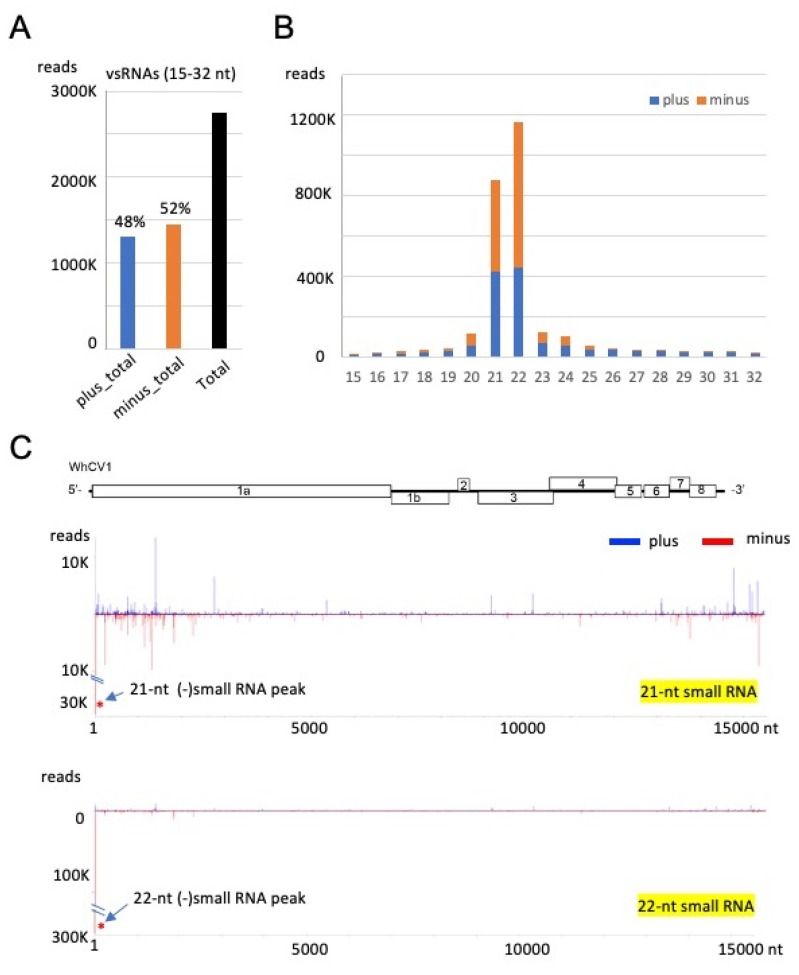
Small RNA profiles of WhCV1 derived from the wheat leaves collected during the 2018/2019 winter wheat-growing season. (**A**) The number of plus (+)- and minus (−)-strand WhCV1-WL19a small RNAs (15–32 nt). (**B**) The size distribution of WhCV1-derived small RNAs (15–32 nt) per million total small RNAs. (**C**) Distributions of 21-nt and 22-nt small RNAs across the entire WhCV1-WL19a genome. The x-axis indicates the map position within the genome and the y-axis shows small RNA read numbers. The (−)-strand small RNA peak mapped to the 3′-terminal end of the WhCV-WL19a genome is indicated with asterisks. (+)-strand small RNA, plus; (−)-strand small RNA, minus.

To characterize WhCV1-derived small RNAs, we performed deep sequencing analysis of the small RNA fraction extracted from the “Chinese Spring” yellow leaf samples collected in the field during the 2018/2019 winter wheat-growing season (Figure 1A). Small RNA reads ranging from 15 to 32 nt in length were mapped onto the WhCV1-WL19a genome (CSL19_contig 5). WhCV1-WL19a-derived viral small RNAs (vsRNAs) accounted for 7.6% (2747884 reads) of the total small RNAs, and the abundance of negative-strand vsRNAs (52%) was slightly higher than that of positive-strand vsRNAs (48%; Figure 5A). The majority of the WhCV1-WL19a vsRNAs were 21- or 22-nt long for both strands and no clear bias toward adenine (A) or uracil (U) for 5ꞌ-terminal nucleotide was observed (Figure 5B and Supplementary Figure S4A). The 21-nt vsRNAs were distributed across the entire WhCV1-WL19a genome regions with several existing hot spots around both the 5′ and 3′ ends of the genome (Figure 5C), whereas, interestingly, the highly abundant 22-nt small RNAs (and the 21-nt small RNAs to a lesser extent) were predominantly distributed in the 3′-terminal end of the WhCV1-WL19a negative-strand genomic RNA (Figure 5C, arrows and Supplementary Figure S4B,C. When a small RNA dataset derived from wheat cv. “Kitahonami” infected with a betaflexivirus [35] or “Chinese Spring” [36] uninfected with WhCV1 was analyzed, no small RNA read was mapped to the 3′-terminal end of the WhCV1-WL19a negative-strand genomic RNA (data not shown), suggesting that the abundant 22-nt small RNAs were specifically derived from WhCV1 RNAs and probably not from the host plant.

## 3. Discussion

In this study, we discovered two molecularly diverse isolates of a closterovirus tentatively named “wheat closterovirus 1 WL19a and WL20” (WhCV1 WL19a and WL20) from yellowing leaf samples of wheat plants in the IPSR experimental field, Kurashiki, Japan, through RNA-seq analysis (Figure 1A and 2). The genome organization of WhCV1 showed the typical features of monopartite closterovirids, including the presence of the putative +1 ribosomal frameshift site for the expression of the RdRP [12]. Phylogenetic relationships based on the HSP70h, RdRP, and CP amino acid sequence showed that WhCV1 was placed in a clade together with the members of the genus *Closterovirus (*Figure 4). The BLAST identities of relevant gene products (RdRP, CP, and HSP70h) shared between WhCV1 and members of known *Closterivirus* species were above the species demarcation criteria (i.e., >25% amino acid sequence divergence) for the genus [12] (Table 2 and Supplementary Figure S3), suggesting that WhCV1, together with TriPCV (a closterovirus candidate identified through transcriptome data mining) [34], represents a member of novel species in the genus *Closterovirus*. We observed that WhCV1-WL20 had aphid transmissibility (a typical feature of closteroviruses) and typical filamentous virion (Figure 3 and Supplementary Figure S2), along with BYDV-PAV coinfection in some cases (Figure 1D and Table 1).

In the early 1970s, a closterovirus named “wheat yellow leaf virus” (WYLV) was recorded to be associated with diseased wheat, barley, rye (*Secale cereale* L.), and oat (*Avena sativav* L.) plants showing yellowing or other leaf symptoms in the IPSR field [10,11]. Considering that the IPSR field is under relatively closed conditions, WYLV populations may have been preserved, meaning that WhCV1 and WYLV may potentially be the same species. However, because the WYLV sample obtained from that period is not currently available, we are unable to verify whether WhCV1 identified in this study is the same virus species as WYLV. WYLV or closterovirus-like agents identified morphologically have been reported in other districts in Japan, as well as in China and Italy, from wheat or other cereals such as Italian ryegrass (*Festuca perennis* L.) [10,11,37,38]. Accordingly, our and other groups’ data-mining of transcriptomes suggests that a WhCV1-related virus may occur in China with dwarf polish wheat as a host [33,34] (Figure 3). Similarly, some short unannotated TSA sequences (accession nos. GDCY01015784, GDCY01015776, and GDCY01015779, 1410 nt, 5655 nt, and 1473 nt, respectively) from ryegrass (*Lolium perenne* L.) infected with an endophyte, *Epichloë festucae* [39], whose predicted partial amino acid sequences are distantly related to WhCV1 replicase, were also detected (data not shown). Thus, a WhCV1 relative, likely a different species, may also be present in Italy, with ryegrass as a host [39]. However, further virus detection assays must be performed to confirm the infection by these viruses.

Many closterovirids have a large +RNA genome (13–19 kb), and their genes are expressed through polyprotein processing, translational frameshifting, and multiple subgenomic RNAs (sgRNAs) [12]. Regarding polyprotein processing, L-Pro (a papain-like cysteine protease) domains in the WhCV1 ORF1a protein have two key catalytic residues (Cys_457_ and His_517_) and one conserved residue (Gly_535_) at the cleavage site [40] (Figure 2A). However, the residue of the potential cleavage site (Met_536_) following Gly_535_ in the WhCV1 ORF1a protein is likely different from that of many other closteroviruses (mainly Gly). Another domain in the ORF1a protein, named the “Zemlya region” (Figure 2A), has three conserved residues (Glu_1237_, Pro_1325_, and Arg_1329_). The latter two residues are likely located within a potential membrane-binding amphipathic α-helix, which could be responsible for the induction of endoplasmic reticulum reconstruction [41]. Additionally, a domain, PHA03247 (E-value = 1.4e^−05^), that is found in the large herpesvirus tegument protein UL3qs6 was also predicted to exist in the N-terminal end region of the WhCV1 ORF1a protein (Figure 2A). This domain is also present in some fungal +RNA viruses in the order *Tymovirales* [42].

The protein products of the second gene unit (a small ORF2 protein, HSP70h, HSP90h [~60 kDa protein], CPm, and CP) of closterovirids are known to be either a major virion or its integral components and are involved in viral cell-to-cell movements [13,25,43]. Like other viruses of the alphavirus-like superfamily, the expression of the second gene unit and downstream ORFs occurs through 3′ co-terminal sgRNAs [12]. Similar to the observation of a CTV isolate [24], mapped reads of WhCV1 were strikingly abundant in the 3′-terminal region, including two 3′ proximal ORFs (ORFs 7 and 8; Figure 2A, lower row), which suggests a high-level transcription of sgRNAs in infected plants. In the closteroviruses, beet yellows virus (BYV) p20, a putative counterpart of the WhCV1 ORF7 protein, is necessary for long-distance movement through the plant vascular system [44], while BYV p21, a putative counterpart of the WhCV1 ORF8 protein, has a function as a VSR [13,45,46]. It has been reported that many wheat- and other cereal-infecting viruses encode VSRs to counteract RNA silencing-mediated antiviral defense systems [47,48].

RNA silencing is an important defense mechanism against viral pathogens. Virus-derived small RNAs accumulate upon virus infection in plants via the processing of double-stranded RNA derived from the virus genome by host dicer-like proteins (DCLs, mainly DCL2 and DCL4) to produce 22- and 21-nt vsRNAs [49,50,51]. In our results, the abundance of WhCV1-derived small RNAs with 21- and 22-nt lengths showed similar characteristics to the vsRNAs of cereal RNA viruses or other closteroviruses [52,53] (Figure 5B). Preferential WhCV1-derived vsRNA accumulation in the 3′-genome region was also reported for the CTV infection; this may presumably be associated with the high transcription of sgRNAs from this region, which has also been suggested based on the results of RNA-seq analyses (Figure 2A) [53]. Interestingly, highly abundant 22-nt-class small RNAs, potentially derived from the 3′-terminal end of the WhCV1 negative-strand genome RNA, were found (Figure 5C and Supplementary Figure S4). This may be due to the formation of highly structured RNA in the terminal end of the genome, but the genomes of other closterovirids are not known to have such a characteristic [15,53,54]. It is tempting to speculate that these small RNAs may possibly act as decoy RNA (small RNA sponge) to counteract antiviral RNA silencing, as previously demonstrated [55,56]. The biological significance of the small RNAs possibly derived from the terminal end of WhCV1 genomic RNA is worthy of further investigation in the future.

In summary, our findings, along with the recent rediscovery of a mealybug-transmitted ampelovirus (sugarcane mild mosaic virus, genus *Ampelovirus* within the family *Closteroviridae*) in sugarcane (*Saccharum* hybrid spp.) [57], have revealed the extent in which closterovirids prevail in wheat and other cereal crop species. These cereal-infecting closterovirids may attract further attention from plant virologists and plant pathologists as some aphid-borne cereal pathogens, as demonstrated by the aphid-transmitted viruses of yellow dwarf cereal diseases, have the potential to cause pandemics or epidemics due to global warming and increasing air temperatures [3,58,59].

## 4. Materials and Methods

### 4.1. Plant Sampling and RNA Extraction

Wheat leaf samples were collected from the experimental field of the IPSR Okayama University, Kurashiki, Japan (34°59′ N, 133°77′ E). Three leaves from each of three symptomatic wheat plants cv. “Chinese Spring” collected in April 2019 (2018/2019 winter wheat-growing season; Figure 1A) were subjected to RNA extraction and followed by RNA-seq and small RNA analyses. The leaves of wheat cv. “Chinese Spring” that showed yellowing, were also collected in mid-May 2019 and stored at −80 °C until analyzed. A total of 12 leaves derived from different wheat plants were subjected to WhCV1-WL19a detection by RT-PCR (Figure 1D). In April 2020 (2019/2020 growing season), three leaves from symptomatic wheat plants cv. “Chinese Spring” were used for the aphid transmission and RT-PCR analyses (Figure 1B,C). All plant samples except for the leaves used for the aphid transmission test were stored at −80 °C until analyzed.

Total RNA from each leaf sample was extracted using the NucleoSpin RNA Plant and Fungi Kit (Macherey and Nagel, Düren, Germany) or TaKaRa RNAiso Plus Reagent (TaKaRa Biotech. Co., Shiga, Japan), following the manufacturer′s instructions. The quantity and purity of RNA were measured with a NanoDrop spectrophotometer (NanoDrop 2000c, Thermo Fisher Scientific, Inc. Waltham, MA, USA). Additionally, the total RNA fraction was analyzed with electrophoresis in a 1% (W/V) agarose gel in 1 × TAE (tris-acetate-ethylenediamine) buffer and stained with ethidium bromide.

### 4.2. Next-Generation Sequencing and De Novo Read Assembly

RNA-seq analysis was basically performed as described previously [60]. Total RNA samples from wheat leaves from the 2018/2019 growing season (228.0 ng/μL; RNA integrity number, RIN = 7.7; Figure 1A) and wheat leaves infected with a laboratory virus WL20 isolate (see below; 60.0 ng/μL; RIN = not measured) were subjected to RNA-seq analysis. After the rRNA depletion using the Ribo-Zero kit (Illumina, San Diego, CA, United States) or QIASeq FastSelect rRNA removal kit (Qiagen, Hilden, Germany), the sample was employed as a template for the cDNA library construction using the TruSeq RNA Sample Preparation kit v2 (Illumina) or MGIEasy FS DNA Library Prep Set (MGI Tech, Shenzhen, China). Subsequently, the respective library was subjected to deep RNA-seq using the Illumina HiSeq 2000 platform (Illumina, paired-end 101 bp reads) or DNBSEQ-G400RS (MGI, paired-end 150 bp reads). rRNA depletion, cDNA library construction, and RNA-seq analysis were performed by Macrogen Japan Co. (Tokyo, Japan) and Genome-Lead Co. (Kagawa, Japan). After adapter and quality trimming of raw reads (the total read number was 64.0 M reads from Illumina sequencing or 18.2 M reads from DNBSEQ sequencing), the sequence reads were de novo assembled using the CLC Genomics Workbench version 11 (CLC Bio-Qiagen, Aarhus, Denmark) with default parameters (length fraction = 0.5; similarity fraction = 0.8). Assembled sequence contigs (total contig number: 81,494 or 51,964) were used as queries for BLAST analyses against the viral genome RefSeq dataset of the NCBI. Sequence reads were mapped to the virus or virus-like contigs using the Read Mapping algorithm with stringent mapping parameters (length fraction = 0.9; similarity fraction = 0.95) in the CLC Genomics Workbench.

### 4.3. RT-PCR and RLM-RACE Analyses of Viral RNA Sequences

For RT-PCR detection, cDNA was synthesized using Moloney-murine leukemia virus reverse transcriptase (Thermo Fisher Scientific) with random hexamers and used as a PCR template with QuickTaq HS Dye Mix (Toyobo, Osaka, Japan). The PCR conditions were as follows: 94 °C for 2 min; 30 cycles of 94 °C for 10 s, 53 °C for 30 s, and 72 °C for 1 or 2 min; and 72 °C for 10 min. The wheat 18S rRNA gene was used as the reference target for the RT-PCR [61]. The primer sets used for virus detection are provided in Supplementary Table S1.

To verify the viral RNA sequences, RT-PCR was performed using sets of overlapping primers. The sequences of the viral primers used in the RT-PCR are available upon request. The PCR-amplified products were directly Sanger-sequenced from both directions or ligated into a TA cloning vector (pGEM-T Easy vector, Promega, Madison, WI, USA) and transformed into *Escherichia coli* strain DH5α (TaKaRa). The plasmid clones were then subjected to DNA sequencing. The 5′- and 3′-terminal sequences of viral RNA were obtained through the 3′RLM-RACE procedure as described by Lin et al. [62]. RLM-RACE amplicons were then subjected to direct DNA sequencing.

### 4.4. Database Search and Sequence Analysis

Sequence data were analyzed using Enzyme X v3.3.3 and 4peaks v1.8 (nucleobytes.com/enzymex/index.html, accessed on 21 October 2022), or the GENETYX-MAC 10.0 and Genetyx Mac ATSQ v3.0 computer programs (Genetyx Co., Tokyo, Japan). BLAST searches were performed using the GenBank database through the NCBI website (nucleotide collection, nr/nt; non-redundant protein sequence, nr; TSA) (https://blast.ncbi.nlm.nih.gov/Blast.cgi, accessed on 1 October 2022). The RNA secondary structures of viral RNA sequences were predicted using the Mfold web server [63] (http://www.unafold.org/mfold/, accessed on 21 October 2022). The conserved domain searches were conducted using the NCBI conserved domain database [64] (https://www.ncbi.nlm.nih.gov/Structure/cdd/wrpsb.cgi, accessed on 21 October 2022). Putative transmembrane domains were predicted using the TMHMM server version 2.0 (https://services.healthtech.dtu.dk/services/TMHMM-2.0/, accessed on 21 October 2022). Pairwise sequence identity comparisons were performed using the Clustal Omega online program of the European Bioinformatics Institute [65] (EMBL-EBI, https://www.ebi.ac.uk/Tools/msa/clustalo/, accessed on 21 October 2022).

### 4.5. Phylogenetic Analysis

The construction of ML trees was basically performed as described previously with minor modifications [66]. Multiple amino acid alignments were constructed using MAFFT online version 7 under default parameters [67] (https://mafft.cbrc.jp/alignment/server/, accessed on 1 October 2022). Unreliable regions of the alignments were removed with TrimAI software version 1.3 in the Phylemon 2.0 online platform with a strict plus setting [68] (http://phylemon2.bioinfo.cipf.es, accessed on 1 October2022). ML trees with 1000 bootstrap replicates were then generated using the PhyML 3.0 online program with automatic model selection by Smart Model Selection [69,70] (http://www.atgc-montpellier.fr/phyml/, accessed on 1 October 2022). The phylogenetic trees (rooting at mid-point) were subsequently visualized using FigTree software version 1.3.1 (nucleobytes.com/enzymex/index.html, accessed on 21 October 2022). The closterovirids that do not belong to the genus *Closterovirus* were used as the distant outgroups in each tree.

### 4.6. Small RNA Analysis

RNA was extracted from the symptomatic wheat leaves (cv. “Chinese Spring”; Figure 1A) collected during the 2018/2019 growing season using TaKaRa RNAiso Plus (609.0 ng/μL; RIN = 7.5) and were subjected to small RNA sequencing. The cDNA library was prepared using the TruSeq Small RNA Library Prep Kit (Illumina), and subsequent deep RNA seqencing was conducted using the Illumina HiSeq 2500 (Illumina, 51-bp single-end reads). The cDNA library construction and deep sequencing were performed by Macrogen Japan. Raw sequence reads (total read number: 36,208,915) were trimmed of adapters and filtered for a size range of 15- to 32-nt lengths using the CLC Genomic Workbench. The retained reads were subsequently mapped onto the virus genome using the Read Mapping algorithm with stringent mapping parameters (length fraction = 1.0; similarity fraction = 1.0) in the CLC Genomic Workbench. The mapped small RNA reads were then subjected to in-depth analysis using the program MISIS-2 [71].

### 4.7. Aphid Transmission Assay

To evaluate the aphid transmissibility of WhCV1, a laboratory aphid colony (*R. padi)* was established from a single adult collected from a barley plant in the IPSR field [72] and reared on virus-free seedlings of barley cv. “Kobinkatagi” in a growth chamber (around 16 °C, 12 h light/12 h dark). Wheat cv. “Chinese Spring” leaf samples showing typical yellow leaf symptoms that were positive for WhCV1-WL20 but not for WhCV1-WL19a or BYDV-PAV (Figure 1C and data not shown) were collected from the field during the 2019/2020 growing season and used as a virus inoculum source. After a 48 h acquisition access period of placing aphids on a symptomatic leaf in a petri dish, 10–15 aphids were placed on one leaf of seedlings of cvs. “Chinese Spring” and “Kihahonami”, followed by a 5-day inoculation access period. Plants were then treated with the insecticide Acephate (Ortran, Sumitomo Chemical Garden Products, Tokyo, Japan). At 4–6 weeks post-inoculation, total RNA was extracted from the wheat plants and then subjected to RT-PCR detection for the systemic virus infection of WhCV1 variants and BYDV-PAV (primers listed in Supplementary Table S1). One virus isolate (WL20) was selected from the infectants, maintained through aphid transmission in the growth chamber as a laboratory WhCV1 isolate, and used for the RNA-seq analysis (see above). Virus particles were negatively stained with an EM stain (Nissin EM Co., Tokyo, Japan) and observed using a model H-7650 transmission electron microscope (Hitachi, Tokyo, Japan).

## Figures and Tables

**Table 1:** Annotated virus or virus-like contigs from RNA-seq of wheat leaves.

Virus-Isolate	Contig or Concatenate	Size (nt)	Total Reads	Accession No.
WhCV1-WL19a	CSL19_contig 5	15,452 (15,145) ^1^	1,995,569	LC735716
-WL20	CSL20_contig 8_12	15,369 ^1^	8,619,360 ^2^	LC735717
BYDV-PAV-WL19b	CSL19_contig 1289	5699	37,336	LC735718

^1^ The entire genomic regions of both viruses were detected using RT-PCR and Sanger sequencing. The termini of WhCV1 genomic RNA were determined using the RACE method, except for the 5′-terminal sequence of the WL20 isolate (CSL20_contig 8_12). The original contig length of CSL19_contig 5 is shown in parentheses. ^2^ The data were obtained with the Read Mapping algorithm.

**Table 2:** The results of BLAST-P analyses using WhCV1-WL19a proteins as queries.

Top Hit Viruses	QC ^1^	E-Value	Identity	Accession
ORF1a protein				
Rehmannia virus	65%	9e^–160^	34.4%	YP_009552004.1
Tobacco virus	64%	2e^–153^	34.6%	YP_009162621.1
Beet yellows virus	47%	2e^–151^	49.8%	AAF14300.1
Cnidium closterovirus	46%	4e^–150^	45.6%	UNN55308.1
Mint virus	46%	3e^–148^	44.6%	YP_224090.1
ORF1b protein (RdRP)				
Soybean leaf crinkle mottle virus	98%	5e^–175^	61.3%	BCR37031.1
Citrus tristeza virus	95%	6e^–169^	60.6%	ABW97532.1
Tobacco virus	97%	9e^–168^	59.3%	YP_009162622.1
Carnation yellow fleck virus	98%	1e^–166^	57.8%	BBK15488.1
Alcea rosea virus	97%	2e^–166^	59.2%	QCW05658.1
ORF3 protein (HSP70h)				
Cnidium closterovirus	99%	0.0	51.4%	UNN55311.1
Carrot closterovirus	99%	0.0	51.3%	AHA85412.1
Carrot yellow leaf virus	99%	0.0	50.5%	AHA85522.1
Thesium chinense closterovirus	99%	0.0	47.2%	UQR78691.1
Mint virus	99%	8e^–177^	46.0%	AAX98726.1
ORF4 protein (HSP90h)				
Mint virus	87%	1e^–140^	43.9%	YP_224094.1
Tobacco virus	94%	2e^–133^	41.7%	YP_009162625.1
Rehmannia virus	87%	1e^–128^	41.9%	YP_009552007.1
Alcea rosea virus	87%	4e^–128^	42.2%	QCW05661.1
Beet yellows virus	90%	2e^–126^	38.4%	S28713
ORF5 protein (CPm)				
Beet yellow stunt virus	84%	8e^–38^	38.0%	YP_009664809.1
Carnation yellow fleck virus	95%	3e^–37^	33.3%	YP_008858535.1
Beet yellows virus	95%	6e^–35^	31.3%	NP_041874.1
Carrot yellow leaf virus	95%	2e^–34^	35.8%	AHA85464.1
Mint virus	91%	1e^–33^	36.6%	YP_224095.1
ORF6 protein (CP)				
Grapevine leafroll-associated virus 2	94%	2e^–16^	33.5%	AHW79716.1
Beet yellow stunt virus	82%	6e^–13^	32.4%	YP_009664810
Carrot closterovirus	91%	9e^–12^	29.3%	AHA85415.1
Carnation necrotic fleck virus	93%	1e^–10^	25.4%	BBK15484.1
Carrot yellow leaf virus	83%	9e^–08^	29.1%	AHA85425.1

^1^ QC: Query Cover.

## Data Availability

The virus and virus-like sequences derived from this study have been submitted to the GenBank/DDBJ/ENA with the accession numbers LC735716–LC735718.

## Supplementary Materials

The following supporting information can be downloaded at: https://www.mdpi.com/article/10.3390/pathogens12030358/s1, Figure S1: The potential secondary structures of the 5′- and 3′-UTRs of the WhCV1-WL19a genome RNA as predicted by Mfold under default settings; Figure S2: The representative virus-like particles observed in the wheat seedling showing mild yellowing leaf symptoms after aphid feeding inoculation (see Figure 3A,B); Figure S3: Pairwise comparison of the HSP70h (A), RdRP (B) and CP (C) encoded by closteroviruses; Figure S4: Profiles of WhCV1-W19a small RNAs.; Table S1: List of primers used in this study.
