# Supplementary material for: Discovery and Genome Characterization of a Closterovirus from Wheat Plants with Yellowing Leaf Symptoms in Japan
Source: Pathogens. 2023 Feb 21;12(3):358. doi: 10.3390/pathogens12030358 (PMC10053543; doi:10.3390/pathogens12030358)
Supplement: Supplementary file 1 [file pathogens-12-00358-s001.zip › pathogens-2110429-supplementary.pdf]

Supplementary Materials:

Discovery and genome characterization of a closterovirus from wheat plants with yellowing leaf symptoms in Japan

Hideki Kondo[1,*], Hitomi Sugahara[1], Miki Fujita[1], Kiwamu Hyodo[1], Ida Bagus Andika[2], Hiroshi Hisano[1], and Nobuhiro Suzuki[1]

[1] Institute of Plant Science and Resources (IPSR), Okayama University, Kurashiki 710-0046, Japan
[2] College of Plant Health and Medicine, Qingdao Agricultural University, Qingdao 266109, China.
* Correspondence: **author Hideki Kondo** Institute of Plant Science and Resources (IPSR), Okayama University, Kurashiki 710-0046, Japan E-mail: hkondo@okayama-u.ac.jp Tel. +81(86) 434-1232 / Fax. +81(86) 434-1232

Supplemental Tables, 1; Supplemental Figures, 4

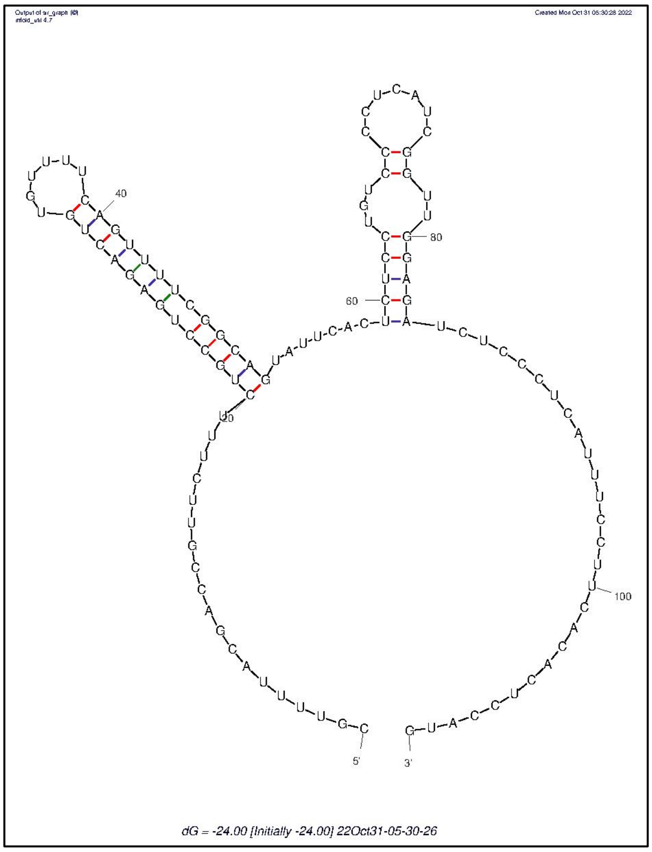

5′ UTR (ΔG = -24.00 kcal/mol)

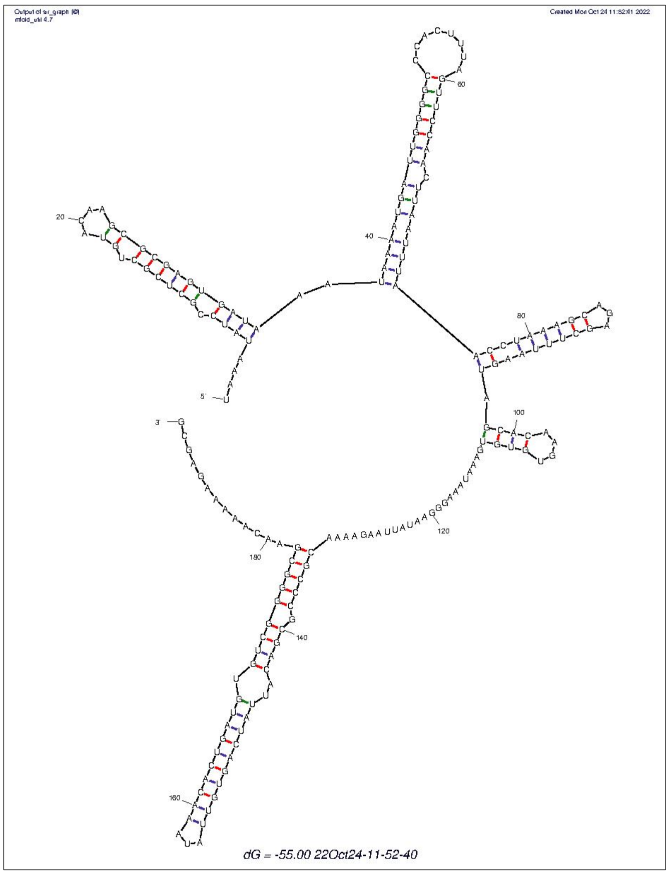

3′ UTR (ΔG = -55.00 kcal/mol)

Figure S1. The potential secondary structures of the 5'- and 3'-UTRs of the WhCV1-WL19a genome RNA as predicted by Mfold under default settings.

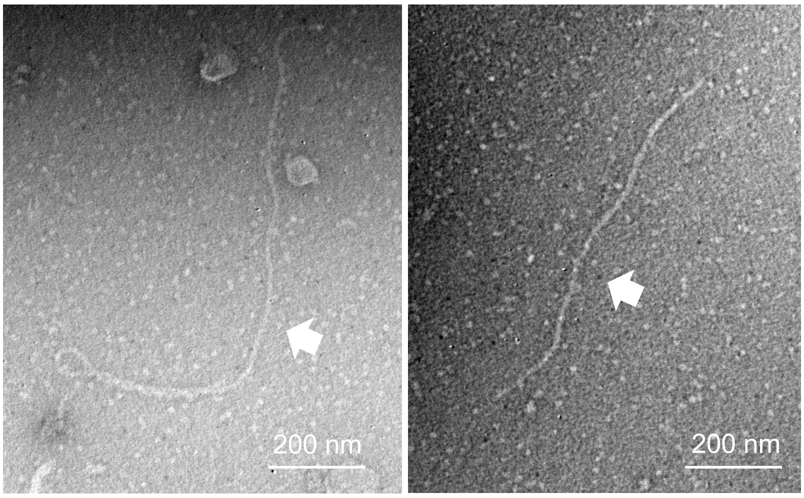

Figure S2. The representative virus-like particles observed in the wheat seedling infected with WhCV1-WL20 showing mild yellowing leaf symptoms after aphid feeding inoculation (see Figure 3A and B). The scale bar is 200 nm.

A

Percent Identity Matrix - created by Clustal Omega

virus name	**HSP70h** accession no.	1	2	3	4	5	6	7	8	9	10	11	12	13	13	14	16	17	18
1 blackcurrant closterovirus 1	YP_009553662	100																	
2 citrus tristeza virus	NP_042864	39	100																
3 raspberry leaf mottle virus	YP_874188	39	45	100															
4 rose leaf rosette-associated virus	YP_009058932	41	48	52	100														
5 strawberry chlorotic fleck-associated virus	YP_762625	38	47	53	52	100													
6 arracacha virus 1	YP_009551996	38	39	40	39	38	100												
7 **WhCV1_WL19a (CSL19_contig 5)**		39	42	41	39	40	42	100											
8 **WhCV1-WL20 (CSL20_contig 8_12)**		39	42	41	39	40	42	98	100										
9 TSA_Triticum polonicum closterovirus	DAZ91134	39	42	41	39	40	42	98	100	100									
10 mint virus 1	YP_224093	37	38	41	42	39	39	46	46	46	100								
11 rehmannia virus 1	YP_009552006	38	40	43	41	39	39	44	44	44	62	100							
12 tobacco virus 1	YP_009162624	37	39	43	41	38	38	43	44	44	66	74	100						
13 carnation necrotic fleck virus	YP_009506334	44	45	49	48	45	43	52	52	52	57	57	56	100					
14 carrot yellow leaf virus	YP_003075968	42	45	45	43	44	43	50	51	51	49	50	50	63	100				
15 carrot closterovirus 1	AHA85412	42	45	45	44	44	42	51	52	52	51	50	48	61	69	100			
16 grapevine leafroll-associated virus 2	YP_337914	39	39	39	37	38	40	42	42	42	40	41	40	46	44	44	100		
17 beet yellows virus	NP_041872	41	40	45	42	40	44	45	45	45	42	47	45	50	48	47	47	100	
18 beet yellow stunt virus	YP_009664807	41	41	40	39	41	43	45	45	45	42	43	42	48	48	48	47	53	100

B

Percent Identity Matrix - created by Clustal Omega

virus name	**RdRP** accession no.	1	2	3	4	5	6	7	8	9	10	11	12	13	13	14	16	17	18
1 carrot yellow leaf virus	YP_003075965	100																	
2 carrot closterovirus 1	AHA85410	84	100																
3 carnation necrotic fleck virus	YP_009506332	61	60	100															
4 mint virus 1	YP_224091	64	63	69	100														
5 rehmannia virus 1	YP_009552003*	65	66	65	82	100													
6 tobacco virus 1	YP_009162622	65	64	67	83	89	100												
7 **WhCV1_WL19a (CSL19_contig 5)**		56	54	56	59	59	59	100											
8 **WhCV1-WL20 (CSL20_contig 8_12)**		56	55	57	58	60	60	88	100										
9 TSA_Triticum polonicum closterovirus	DAZ91132*	55	55	57	58	59	59	89	99	100									
10 rose leaf rosette-associated virus	YP_009058929	54	54	54	55	54	55	55	55	55	100								
11 citrus tristeza virus	NP_042860	55	54	56	58	56	58	58	58	58	57	100							
12 raspberry leaf mottle virus	YP_874185	55	54	53	56	55	55	58	59	58	62	64	100						
13 strawberry chlorotic fleck-associated virus	YP_762622	56	57	56	56	58	57	58	58	58	61	65	65	100					
14 blackcurrant closterovirus 1	YP_009553660*	52	50	52	52	52	52	52	52	52	50	53	54	52	100				
15 grapevine leafroll-associated virus 2	YP_337912	55	54	56	56	57	56	55	54	54	52	55	55	55	56	100			
16 arracacha virus 1	YP_009551994	55	55	55	58	57	59	55	55	55	51	56	55	56	58	61	100		
17 beet yellows virus	NP_041870*	58	56	57	58	56	57	57	55	56	55	58	57	59	55	60	64	100	
18 beet yellow stunt virus	YP_009664804	56	55	53	56	56	57	58	57	57	53	58	57	59	56	62	65	66	100

C

Percent Identity Matrix - created by Clustal Omega

virus name	**CP** accession no.	1	2	3	4	5	6	7	8	9	10	11	12	13	13	14	16	17
1 citrus tristeza virus	NP_042867	100																
2 strawberry chlorotic fleck-associated virus	YP_762628	29	100															
3 rose leaf rosette-associated virus	YP_009058935	25	31	100														
4 raspberry leaf mottle virus	YP_874191	34	31	40	100													
5 blackcurrant closterovirus 1	YP_009553664	24	26	21	24	100												
6 mint virus 1	YP_224096	27	22	21	26	19.58	100											
7 rehmannia virus 1	YP_009552009	24	20	23	28	22.22	48.26	100										
8 tobacco virus 1	YP_009162627	23	21	23	26	22.22	48	63	100									
9 carrot yellow leaf virus	YP_003075971	27	23	27	25	26.29	35.2	35	34	100								
10 carrot closterovirus 1	AHA85415	26	24	24	29	21.65	36.92	39	36	46	100							
11 **WhCV1_WL19a (CSL19_contig 5)**		19	20	20	24	25.81	26.04	25	24	25	27	100						
12 **WhCV1-WL20 (CSL20_contig 8_12)**		19	21	21	24	24.73	26.04	26	24	24	26	95	100					
13 TSA_Triticum polonicum closterovirus	DAZ91137	19	21	21	24	24.73	26.04	26	24	24	26	95	99	100				
14 arracacha virus 1	YP_009551999	22	19	20	25	15.05	27.75	25	29	22	31	28	27	28	100			
15 grapevine leafroll-associated virus 2	YP_337917	23	23	22	23	22.92	23.71	28	29	26	28	29	29	29	35	100		
16 beet yellows virus	NP_041875	23	21	24	25	17.95	28.5	27	27	24	31	25	26	26	31	36	100	
17 beet yellow stunt virus	YP_009664810	23	25	27	28	20	32.99	32	30	27	29	28	28	28	31	34	36	100

Figure S3. Pairwise comparison of the HSP70h (**A**), RdRP (**B**) and CP (**C**) encoded by closteroviruses.

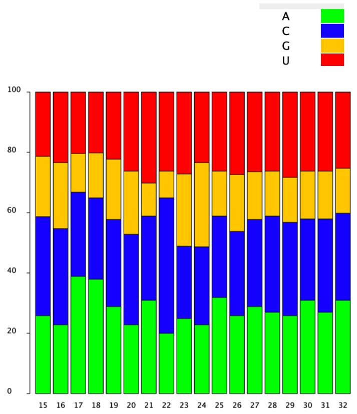
A
C
G
U
100
80
60
40
20
0
15 16 17 18 19 20 21 22 23 24 25 26 27 28 29 30 31 32

A

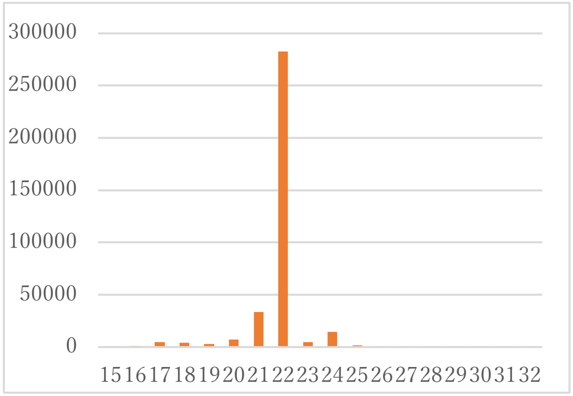
300000
250000
200000
150000
100000
50000
0
15 16 17 18 19 20 21 22 23 24 25 26 27 28 29 30 31 32

C

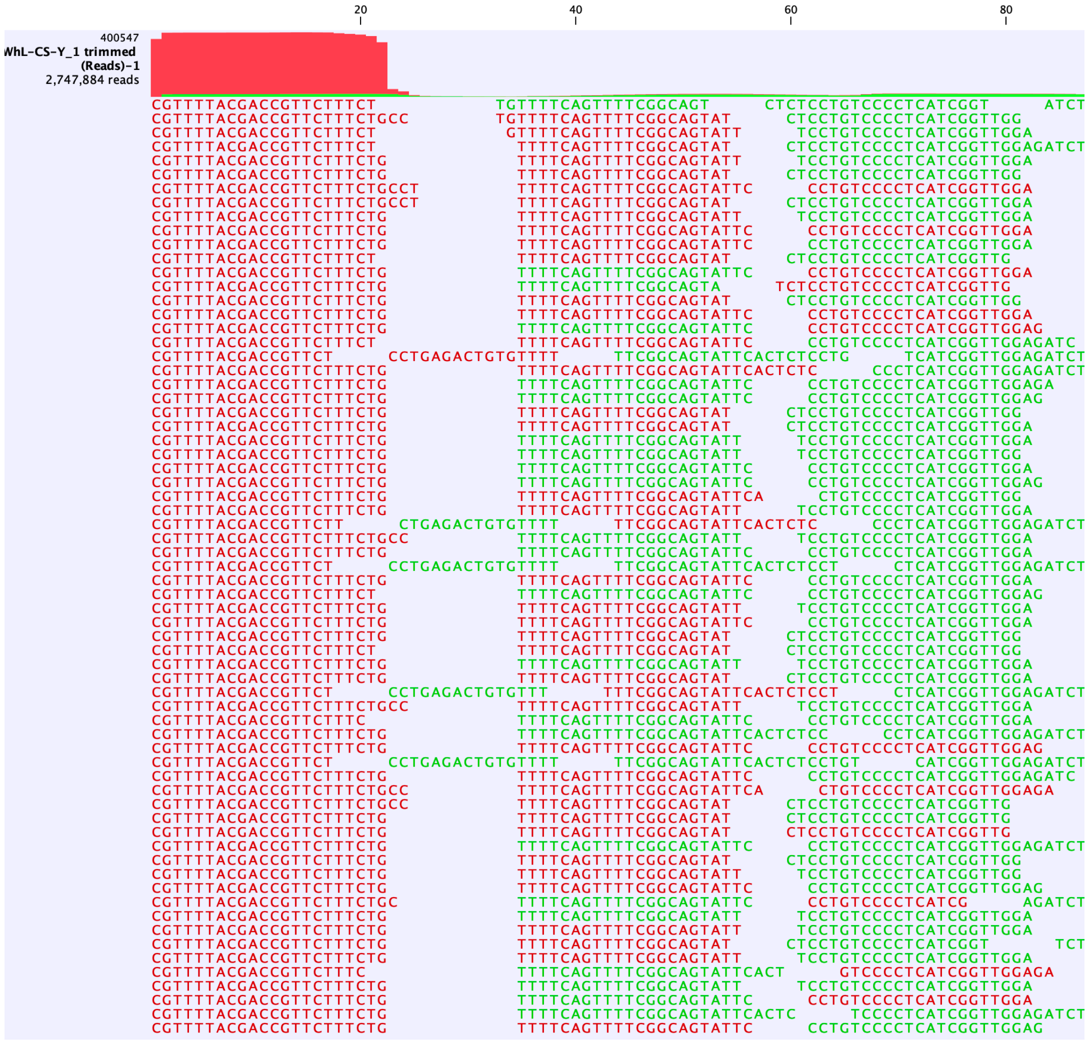
20
40
60
80
400547
WhL-CS-Y_1 trimmed
(Reads)-1
2,747,884 reads

B

Figure S4. Profiles of WhCV1-WL19a small RNAs. **(A)** Proportions of 5'-terminal nucleotide sequences of WhCV1-WL19a-derived small RNAs accumulated in the wheat leaves. **(B)** The partial mapping result of small RNAs at the position around the end of the 5'-terminal region of the WhCV1-WL19a genome. A high number of minus-strand small RNAs were mapped around the nucleotide positions 1–22 of the genome (red text). Small RNA reads were mapped to the WhCV1-WL19a isolate using the Read Mapping algorithm in the CLC Genomics Workbench with stringent mapping parameters (length fraction=1.0; similarity fraction=1.0). **(C)** Size distribution of small RNAs mapped to the nucleotide positions 1–22 of the genome (mostly minus-strand molecules).

Table S1. List of primer used in this study.

Name	oligo nucleotide sequence (5’ → 3’)
detection (WhCV1 variant; WL19a, CSL19_contig 5)	
clost3_c11_F	TGTTGATCCTATACCTAGCCATGCT
clost3_c11_R	GCTGCATAAACACTCTTAAGTGGCC
detection (WhCV1 variant; WL20, CSL20_contig 8_12)	
clost2_c9_F	AACCGATCCCCAGCCACGCCGAAC
clost2_c9_R	TACCCTCTAAGAGGAATGATCCTCG
detection (BYDV-PAV; WL19b, CS19L contig 1289)	
BYDV-PAV_2939F	CACACGAAGGCGGCGCCGTCGAGC
BYDV-PAV_3358R	GCTGCGCGGAAGGACTTTGAGGCG
3’ RLM-RACE (5’ terminal)	
Clo3_CS19L-5RaR1	GGGACCGGCGCAAGTGGTGCGGTG
Clo3_CS19L-5RaR3	AAGCGGTCAGGAGCGGTGTGACGAG
3’ RLM-RACE (3’ terminal)	
Clos2a-Ra3F1	GTTGCGCTGATACGGCGGTCGAG
Clos2a-Ra3F3	CGCTCGCTGTATAATCGCGAGCG
3’ RLM-RACE (adapter and reverse primers)	
5'-p-Adapter	CAATACCTTCTGACCATGCAGTGACAGTCAGCATG
1st-3Race	ATGCTGACTGACACTGCAT
2nd-3Race	CACTGCATGGTCAGAAGGTATT
